# Designing active breaks in secondary school, results from focus group with teachers: the brave study

**DOI:** 10.1093/eurpub/ckac131.448

**Published:** 2022-10-25

**Authors:** G Longo, A Masini, M Ricci, S Marini, A Sansavini, LM Scheier, A Ceciliani, L Dallolio

**Affiliations:** 1 Department of Biomedical and Neuromotor Sciences, University of Bologna, Bologna, Italy; 2 Department for Life Quality Studies, University of Bologna, Rimini, Italy; 3 Department of Psychology Renzo Canestrari, University of Bologna, Bologna, Italy; 4 LARS Research Institute, Inc., Scottsdale, USA

## Abstract

**Background:**

Many adolescents are unable to accomplish the daily 60 minutes of moderate to vigorous physical activity (PA) recommended by WHO. Secondary school is a critical period for developing healthy habits and PA interventions have the potential to promote healthy development. Active Breaks (ABs) are a school-based intervention consisting of short bursts (5-15 minutes) of PA made part of the academic routine. Primary school has been ABs main setting, while secondary school interventions received less attention. The aim of the BRAVE Study is to investigate teachers’ opinion about the feasibility of ABs in Italian secondary school.

**Methods:**

In November 2020 20 teachers from two secondary schools located in Bologna province (Italy) were enrolled in two focus groups (FGs). FGs were held online, recorded, and transcribed. Questions were asked about the role of PA in the school routine and related experiences, perceived barriers and facilitators of the intervention, suggestions regarding the intervention design. Final expectations were then discussed.

**Results:**

Despite limited experience with PA interventions, participants felt ABs would improve psycho-physical well-being for both students and teachers. Lack of time and space and a wary attitude towards ABs were listed as barriers. Program flexibility regarding content, administration time frames and implementation mode was listed as a great facilitator: program should be adaptable to participants’ needs, favoring easy and quick exercises. Overall, expectations emphasized improving classroom behavior and promoting healthy habits.

**Conclusions:**

Teachers felt that inclusion of ABs in secondary school was promising and could lead to many health benefits. ABs were deemed feasible given their short duration and adaptability, since the program can be implemented with current personnel resources and space configurations. Co-design is essential to overcome personal barriers and create an effective and sustainable intervention.

**Key messages:**

• According to secondary school teachers, PA interventions have the potential to improve psycho-physical well-being and classroom environment, promoting healthy habits among students.

• ABs are deemed as a feasible and sustainable PA intervention thanks to program flexibility regarding contents, administration time frames and implementation mode.

